# Surgical Diagrams

**Published:** 1843-02

**Authors:** 


SURGICAL DIAGRAMS.

Dr. Gross, the Professor of Surgery in the Louisville Medical Institute, has just had completed, at his private expense, a series of magnified and colored drawings, which will add greatly to the interest and practical value of the future prelections from his chair. There are one hundred and fifty figures comprised in fourteen colored plates, illustrating the *diseases of the urinary and genital organs; diseases of the rectum; aneurism and diseases of the veins; diseases of the eye; dislocations of the hip-joint, and the manner of reducing them; hernia; polypus of the nose; diseases of the mammary gland; inflammation, abscess, mortification; malignant pustule; erysipelas; cancer of the lip; fungus hematodes; club-foot; hare-lip; and syphilitic diseases of the skin.* These drawings are made on a large scale, so as to be seen from any part of the room, and are executed in the highest style of art. Of their value we need not speak more particularly; it will be readily appreciated by those for whose benefit they are intended. On the whole, they render the material for instruction in this department quite as ample as that of any other in this richly endowed institution.

These figures were drawn and colored—partly from copies, partly from nature—by Mons. A. Suminski, a gentlemanly and accomplished Pole, who has sought refuge in this country from the persecutions of those who lord it over his native land. His merit as an artist is of the very first order. He draws with wonderful accuracy and rapidity, and his coloring is as perfect as art can make it. If any of our brethren, here or elsewhere, wish to be supplied with matters of this kind, we can recommend Mr. S. to them with great confidence.

